# Development of an Atomic-Oxygen-Erosion-Resistant, Alumina-Fiber-Reinforced, Fluorinated Polybenzoxazine Composite for Low-Earth Orbital Applications

**DOI:** 10.3390/polym15010112

**Published:** 2022-12-27

**Authors:** Leah Oppenheimer, Malavika Ramkumar, Irlaine Machado, Chris Scott, Scott Winroth, Hatsuo Ishida

**Affiliations:** 1 Department of Macromolecular Science and Engineering, Case Western Reserve University, 10900 Euclid Avenue, Cleveland, OH 44106-7202, USA; 2 Department of Chemistry, Shiv Nadar University, Tehsil Dadri 201314, UP, India; 3 School of Chemistry, Universidade Federal do Rio de Janeiro, Cidade Universitária, Rio de Janeiro 21941-909, RJ, Brazil; 4 Material Answers LLC, 66 Buckskin Drive, Weston, MA 02493, USA

**Keywords:** benzoxazine, fluorinated benzoxazine, polybenzoxazine, high-performance polymer, atomic-oxygen erosion

## Abstract

An atomic-oxygen-erosion-resistant fluorinated benzoxazine resin and composite were developed. The benzoxazine resin, abbreviated as “BAF-oda-fu,” consists of four benzoxazine rings, and was synthesized from bisphenol AF (BAF), 4,4′-oxydianiline (oda), furfurylamine (fu), and paraformaldehyde. The resin was characterized by infrared spectroscopy (FT-IR), proton nuclear magnetic resonance spectroscopy (^1^H NMR), differential scanning calorimetry (DSC), and thermogravimetric analysis (TGA). An analysis of the solvent-washed product showed a technical grade purity (>95%) and a yield of approximately 85%. Subsequent polymerization of the resin was successfully performed by heating step-wise and opening the benzoxazine rings to form a crosslinked network. Thermal analyses showed a melting temperature of 115 °C and polymerization temperature of 238 °C, both being characteristic values of benzoxazine monomers. The benzoxazine resin was also blended with polyoctahedral sisesquoxane (POSS) and reinforced with alumina fibers. The Tg of the resin, as determined by DMA of the composite, could reach as high as 308 °C when post-curing and the POSS additive were utilized. The low-Earth orbit atomic-oxygen erosion rate was simulated by an RF plasma asher/etcher. The atomic-oxygen resistance of poly(BAF-oda-fu) fell along an established trend line based on its fluorine content.

## 1. Introduction

Polybenzoxazines are a type of thermoset polymer synthesized from a phenol, an amine, and an aldehyde, which react to form a characteristic oxazine ring fused to an aromatic ring. Benzoxazines polymerize via cationic ring-opening polymerization (ROP) based around the oxazine ring. These polymers have many beneficial properties, most notably their thermal stability, seen in their high char yield and glass transition temperature [1,2]. Benzoxazines are also chemically stable and highly tailorable through reagents, making them suitable for numerous advanced applications. Taking advantage of the versatile molecular design flexibility of benzoxazines, polybenzoxazines specifically designed for space applications have been reported [3,4,5,6]. The aim of the current paper was to develop a polybenzoxazine-based composite system that is resistant to atomic-oxygen erosion for low-Earth orbital applications.

Due to the well-known high thermal stability of polybenzoxazines, and the unique ability of fluorinated polymers to resist atomic-oxygen erosion, a fluorinated polybenzoxazine will be used for this application. The synthesis of fluorinated benzoxazines requires rather different conditions from the ordinary, non-fluorinated benzoxazines due in part to the strong electron-withdrawing tendency of the CF group. Fluorinated benzoxazines have been reported in the literature [7,8,9,10,11,12,13,14,15,16,17,18,19,20,21,22,23,24,25,26,27,28,29,30,31,32,33,34,35,36]. Polybenzoxazines derived from fluorinated monomers are studied for their special synthesis methods [8,15,23], low dielectric constant properties [9,10,19,27,29,32,34], structure–property relationships [7,11,28,30], fuel cell applications [13], low surface free energy or superhydrophobicity [12,14], and high thermal properties [16,17,18,20,21,25,31,35,36]. All of these papers attest to the unique advantages of fluorinated polybenzoxazines.

Low-Earth orbit is an environment rich with atomic oxygen, which is highly reactive and can erode or degrade most materials it comes into contact with [37]. Common materials that are used to resist atomic-oxygen erosion include metals and their alloys, such as aluminum, titanium, magnesium, molybdenum, tantalum, and tungsten, as well as polymers such as Kapton (a polyimide), polytetrafluoroethylene, polysulfone, and polyetheretherketone [38,39]. Oftentimes, materials are used as composites, with ceramic (e.g., silica or alumina) reinforcements or coatings to enhance erosion resistance. While atomic-oxygen resistance is the primary concern for materials in low-Earth orbit, these materials must also be able to withstand debris and thermal cycling, while also being lightweight to be effective in spacecraft [37,38]. Polymers, though not as atomic-oxygen-resistant, are generally less dense than metals, and are thus advantageous materials for low-Earth orbit. Previous studies have explored combinations of inherently resistant polymers with protective coatings [39]; however, fluorinated polybenzoxazines are a promising outlook into developing atomic-oxygen resistance without the use of metals.

To further improve the lightweight requirement, this project included the formation of a composite with this benzoxazine and alumina (Al_2_O_3_) fibers to enhance its mechanical strength, as well as provide some atomic-oxygen resistance. Polyhedral oligomeric silsesquioxane (POSS) was also added to the fluorinated benzoxazine resin to further improve the erosion resistance, though the fluorinated benzoxazine structure on its own provides considerable resistance.

The benzoxazine was synthesized from bisphenol AF (BAF), 4,4′-oxydianiline (oda), and p-formaldehyde, with furfurylamine (fu) as a reactive terminal group. Bisphenol AF is a fluorinated bisphenol, containing two CF_3_ groups on the quaternary carbon between aromatic rings. 4,4′-oxydianiline is a diamine commonly used in benzoxazines, though rarely as a bridging compound for a benzoxazine of this size. Many benzoxazines containing oda have high mechanical properties, including tensile strength and Young’s modulus. [40] While static and dynamic mechanical properties may be secondary considerations in materials for low-Earth orbital applications, it is still necessary that the materials be mechanically stable; hence, it is beneficial to use reagents that will enhance the strength of the resin. Furfurylamine, used as the terminal group, contains furan, which contributes to the enhanced crosslink density and thus improves thermal properties, including the glass transition temperature, of the crosslinked system [41]. FU-containing benzoxazines have also been found to have fairly low coefficients of thermal expansion, which is a necessary property for low-Earth orbital applications so that the mechanical stability is retained in the harsh thermal conditions that will be experienced by this resin [41].

## 2. Materials and Methods

### 2.1. Materials

Bisphenol AF (>98%), 4,4′-oxydianiline (>98%), and 1-(2-furyl)methylamine (99%) were purchased from TCI America (Portland, OR, USA). Paraformaldehyde (>95%), tetrahydrofuran (≥99.9%), and TLC Silica gel 60 F254 (100 glass plates, 2.5 cm × 7.5 cm) were obtained from Sigma Aldrich (Burlington, MA, USA). Chloroform and sodium hydroxide (NaOH) were received from Fisher Scientific Company (Waltham, MA, USA). Deuterated chloroform (CDCl3) (99.8%) was purchased from Cambridge Isotope Laboratories., Inc. (Tewksbury, MA, USA). Glycidyl isobutyl POSS (EP0418) was purchased from Hybrid Plastics Inc. (Hattiesburg, MS, USA). Woven 3M Nextel 312 ceramic fabric (Style AF-40, 5 harness satin weave, 1800 denier, heat cleaned) was purchased from Bristal Metal Products, Inc. (Westlake, OH, USA). All chemicals were used as received.

### 2.2. Monomer Synthesis

The benzoxazine monomer, abbreviated as BAF-oda-fu, was synthesized from bisphenol AF, 4,4′-oxydianiline, furfurylamine, and p-formaldehyde in stoichiometric ratios of 2:1:2:8, respectively. The reagent masses used were 168.588 g (0.501 mol) BAF, 50.204 g (0.251 mol) oda, 48.692 g (0.517 mol) fu, and 66.444 g (2.21 mol) p-formaldehyde. The synthesis was performed in a round-bottomed flask for 12 h at 90 °C in chloroform. The completion of the reaction was monitored using thin-layer chromatography (TLC) with chloroform as the solvent system. Following completion of the reaction, the chloroform was evaporated under air for 10 days, after which the product was moved to a vacuum oven to continue drying at 50 °C for 11 h. Once dry, the product was washed thrice with 1N aq. NaOH solution and four times with distilled water to remove unreacted BAF and oda. The washed product was then dried under air. Following this, the product was further purified via column chromatography, using chloroform as the eluting solvent and confirmed using TLC (with the same solvent system). The purified compound was once again dried under air for 5 days to yield the pure, dry, and solid monomer, which was a light-yellow color. The yield before purification was approximately 92%, and 85% (relative to the initial predicted synthetic yield) after purification. Recrystallization was not attempted as a large amount of monomer was needed for subsequent experiments, though column chromatography is typically the primary method of purification for fluorinated benzoxazines.

### 2.3. Preparation of BAF-oda-fu with POSS Additive

A sample of BAF-oda-fu resin with 3 wt% glycidyl isobutyl POSS additive was prepared for use in composite fabrication. A 50 wt% solution of BAF-oda-fu in tetrahydrofuran (THF) and a 10 wt% solution of POSS in THF were prepared in separate glass beakers. The solutions were mixed such that when the THF was evaporated the final ratio was 3 wt% POSS to 97 wt% BAF-oda-fu. The resulting solution was then cast and allowed to dry at room temperature. The thin layer of dried resin (abbreviated as BAF-oda-fu-POSS) was scraped from the pan and crushed into coarse granules.

### 2.4. Polymerization Procedure

**Figure 1:**
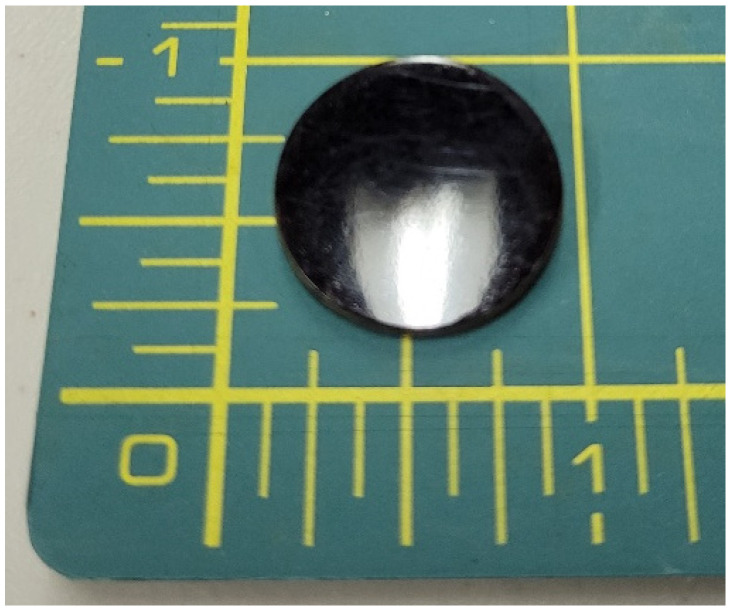
Photograph of poly(BAF-oda-fu) sample. Scale units are inches.

Three methods were adopted to obtain the polymer. The first method involved heating the monomer in a furnace for 2 h at approximately 235 °C. A multiple-step polymerization process was also performed. The sample was heated using a convection oven for half an hour each at 180, 200, 220, and 240 °C. For the third method, polymer specimens were prepared from BAF-oda-fu benzoxazine resin under vacuum by heating for 2 h at approximately 200 °C. A photograph of a poly(BAF-oda-fu) sample prepared using the third polymerization method is provided in Figure 1.

### 2.5. Preparation of Composite Samples

Composite samples, in the form of flat rectangular plates, were fabricated using a low-pressure vacuum bagging process. A hand lay-up process was used to apply materials to the working surface of the mold. Swatches of Nextel 312 AF-40 fabric, 10 cm × 10 cm in size, were used as the composite reinforcement. Two different benzoxazine resin formulations were used as the composite matrix material: BAF-oda-fu resin and BAF-oda-fu with 3 wt% POSS additive.

Swatches of Nextel 312 AF-40 fabric were cut from a larger roll of fabric using a laser cutter (Model QX-80, Rabbit Laser USA, Middletown, OH, USA) with an 80 W, 10.6 μm CO_2_ laser. Optimal results were achieved using 60% laser power over a cut speed range of 5–8 mm/s. The laser cutter was found to provide several benefits over manual cutting tools, including: faster cuts, straighter and more accurate cuts, reduced preparation time (due to elimination of physically measuring and marking cut lines), and almost no edge fraying.

Consolidation and polymerization of all composite samples was achieved by simultaneously applying vacuum and heating the surface of the mold. Prior to starting the polymerization cycle, the vacuum bag mold assembly was held under vacuum for 24 h at room temperature to remove residual moisture from the resin. The mold temperature was then increased from room temperature to approximately 150 °C and held for about 30 min to remove volatiles and allow the resin to melt and wet out the Nextel fabric. The temperature was then increased to 200 °C and held for 2 h.

**Figure 2:**
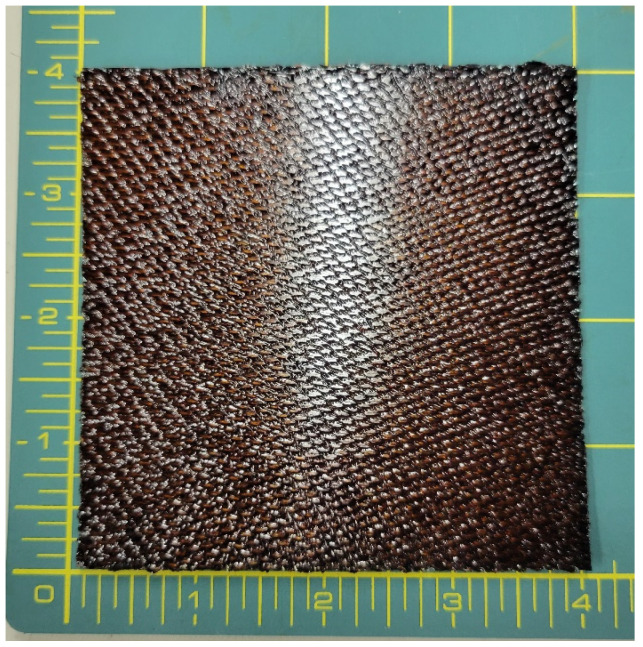
Photograph of a typical 10 cm × 10 cm (4 in × 4 in.) composite sample after post-cure heat treatment for 30 min at 240 °C. Scale units are inches.

For some composite samples, a post-cure heat treatment was performed after the polymerization cycle. Composite samples were heated for 30 min at 240 °C using either a convection oven or hot plate. A photograph of a typical heat-treated composite sample is provided below in Figure 2.

### 2.6. Characterization

^1^H NMR spectra were collected in deuterated chloroform (CDCl_3_) with tetramethylsilane as an internal standard on a Varian Oxford AS600 with a proton frequency of 600 MHz. When a quantitative measurement was to be conducted, a contact time of 10 s was used. Fourier transform infrared spectroscopy (FT-IR) spectra were collected on an ABB Michelson MB3000 FT-IR spectrometer. The spectrometer was equipped with a deuterated triglycine sulfate (DTGS) detector and a dry air purge unit. The absorbance mode used to obtain the spectra was 32 coadded scans at a resolution of 4 cm^−1^. The monomer samples were coated on a 25 mm KBr disk from a chloroform solution. Samples of poly(BAF-oda-fu) isothermally heated at 180, 200, 220, 230, 240, 260, and 280 °C, consecutively, and for 0.5 h at each temperature, were ground with KBr powder and compressed into disks using a hydraulic press for FT-IR analysis. The differential scanning calorimetry (DSC) study was conducted using a TA Instruments DSC model 2920 with a heating rate of 10 °C/min and a nitrogen flow rate of 60 mL/min in the temperature range from room temperature to 300 °C. Thermogravimetric analysis (TGA) was conducted using a TA Instruments model 2950 TGA with a heating rate of 10 °C/min and a nitrogen flow rate of 60 mL/min in the temperature range from room temperature to 800 °C. The flammability of polymer samples was evaluated by microscale combustion calorimetry that was manufactured by Fire Testing Technology, East Grinstead, UK. The heating rate adopted was 1 °C/s from room temperature to 800 °C. Three measurements on the same material were repeated to test the reproducibility of the results. The dynamic mechanical properties of composite samples were measured using a DMA Q800 dynamic mechanical analyzer (TA Instruments, New Castle, DE, USA). Composite specimens with nominal dimensions of 60 mm × 12 mm × 1 mm were cut from composite plate samples using a laser cutter. DMA specimens were tested at a constant strain amplitude of 0.1%, constant frequency of 1 Hz, and heating rate of 5 °C/min. Load was applied using a dual cantilever fixture. For the non-heat-treated specimen, the maximum test temperature was 300 °C. For heat-treated specimens, the maximum test temperature was 450 °C. The density of polymer and composite materials was evaluated in accordance with ASTM D792 (Standard Test Methods for Density and Specific Gravity (Relative Density) of Plastics by Displacement).

^1^H NMR (600 MHz, CDCl_3_) δ 7.40–7.29 (m, 1H), 7.16–6.56 (m, 13H), 6.41–6.05 (m, 3H), 5.30 (d, *J* = 6.7 Hz, 4H), 4.96–4.73 (m, 3H), 4.65–4.40 (m, 4H), 3.97 (d, *J* = 8.4 Hz, 3H), 3.94–3.81 (m, 3H), 1.85–0.72 (m, 1H). FT-IR (cm^−1^) 2910, 1500, 1238, 1190, 1164, 1121, 1078, 962, 929, 817, 733.

## 3. Results and Discussions

### 3.1. Synthesis and Characterization of BAF-oda-fu 

In order to determine the optimum reaction time to achieve the highest conversion of reagent to product, the molar ratios were calculated for each reaction condition using the following equation based on preliminary ^1^H NMR spectra:

(1) $$I_{x}I_{y}N_{y}N_{x}=(M_{x}M_{y})$$

where I_x,y_ refers to the integration value of a given NMR resonance and a reference peak, N_x, y_ refers to the estimated number of protons correlating to the given and reference peaks. M_x_/M_y_ is the molar ratio. From this calculation, it was determined that a reaction time of 12 h yielded molar ratios that were closest to the expected ratio for each resonance. 

**Scheme 1:**
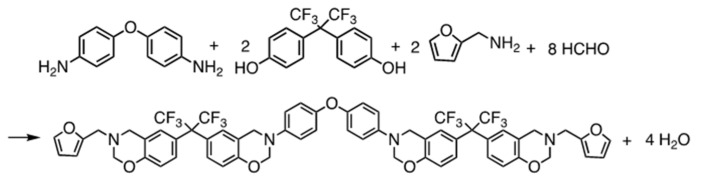
Synthetic scheme of BAF-oda-fu.

Scheme 1 shows the synthetic route used to obtain the required benzoxazine monomer, BAF-oda-fu. As can be seen from the diagram, the product is a symmetric molecular structure containing four benzoxazine units. 

**Figure 3:**
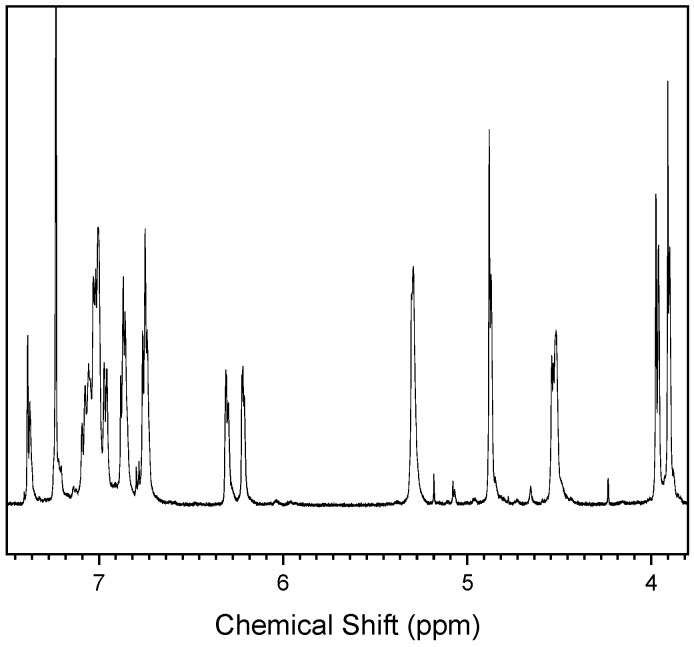
^1^H NMR spectrum of BAF-oda-fu.

Upon completing the successful synthesis, the chemical structure of the monomer was confirmed using ^1^H NMR spectroscopy. The proton NMR spectrum of purified BAF-oda-fu is shown in Figure 3. Resonances located at 5.31 ppm, 4.89 ppm, 4.55 ppm, and 4.00 ppm correspond to the four oxazine(-C_2_H_2_NCH_2_OC-) environments. There are two pairs of oxazine groups that are magnetically equivalent, and each oxazine ring has two CH_2_ groups that are magnetically distinctive; thus, four oxazine CH_2_ resonances are expected. If the product only contained benzoxazine monomers, rather than a mixture of monomers, dimers, and oligomers, then it would be expected to see four singlets corresponding to the oxazine protons, as dimerization would alter the chemical environment. The presence of these singlets indicates the successful synthesis of monomers with purification by column chromatography. The resonances at 5.31 ppm and 4.89 ppm correspond to the protons between the nitrogen and oxygen (-NCH_2_O-), respectively, on the oxazine ring formed from the oda and furfurylamine. Likewise, the resonances at 4.55 ppm and 4.00 ppm correspond to the protons between the nitrogen and aromatic rings (-NCH_2_Ar-) on those same respective oxazine rings. Three resonances are also identified at 7.39 ppm, 6.32 ppm, and 6.25 ppm, which correspond to the three protons on the furan (C_2_H_3_O) group. The resonance located at 3.93 ppm corresponds to the methylene group (-CH_2_-) from the furfurylamine. Several resonances located in the range of 6.70–7.20 ppm were also identified and determined to correlate with the aromatic protons. There is a particularly tall resonance located at 7.26 ppm, which corresponds to excess unevaporated chloroform that remained in the sample in trace amounts.

**Figure 4:**
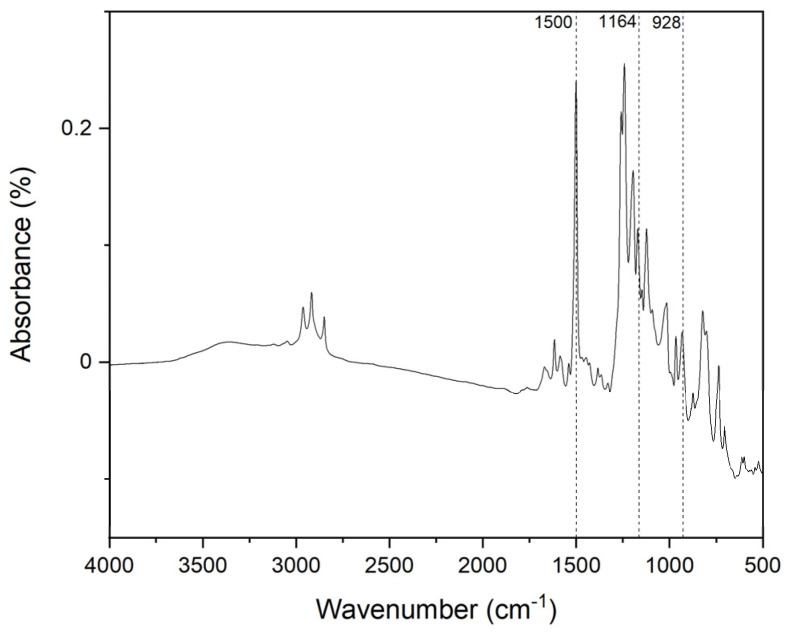
FT-IR spectrum of BAF-oda-fu.

FT-IR was used to further characterize the chemical structure of the product, shown by the spectrum in Figure 4. Characteristic oxazine bands are located at 1238 cm^−1^ (C-O-C antisymmetric stretching), 1121 cm^−1^ (C-O-C symmetric stretching), 961 cm^−1^, and 928 cm^−1^. Bands corresponding to the furan group are located at 1078 cm^−1^ (C-O stretching) and 733 cm^−1^ (furan wagging). The CH bands for benzene are located at 1500 cm^−1^ and 816 cm^−1^, which represent trisubstituted and disubstituted benzene, respectively. The band at 2910 cm^−1^ is indicative of methylene (C-H stretching) from the furfurylamine. A band corresponding to the C-N stretching is located at 1028 cm^−1^. The C-F stretching mode of the CF_3_ group is located at 1164 cm^−1^.

### 3.2. Polymerization Behavior of BAF-oda-fu

**Figure 5:**
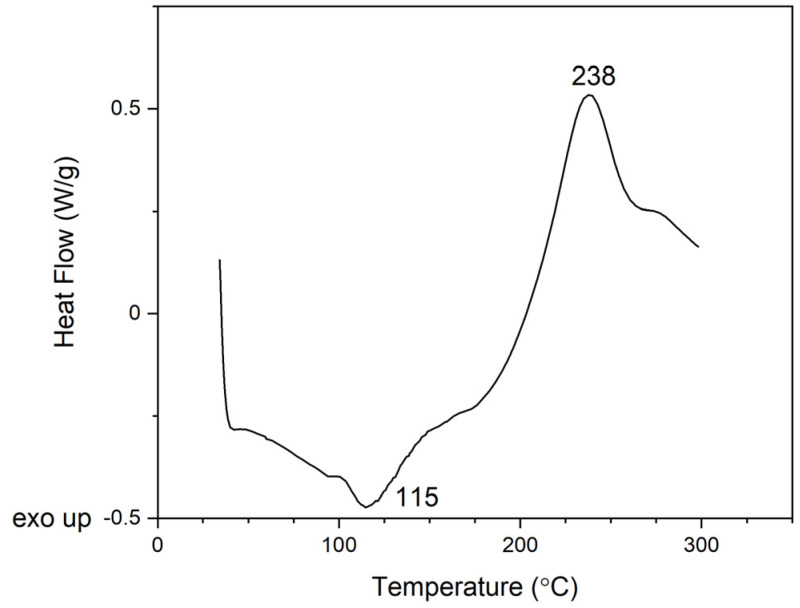
DSC thermogram of BAF-oda-fu.

The polymerization behavior of BAF-oda-fu was studied using DSC and FT-IR analyses. The DSC thermogram is given in Figure 5, where a single exothermic peak is seen at 238 °C corresponding to the polymerization temperature (T_p_) of the resin. The temperature obtained here was later used to determine the heat-catalyzed polymerization temperature. The endothermic peak at approximately 115 °C corresponds to the melting point (T_m_).

**Figure 6:**
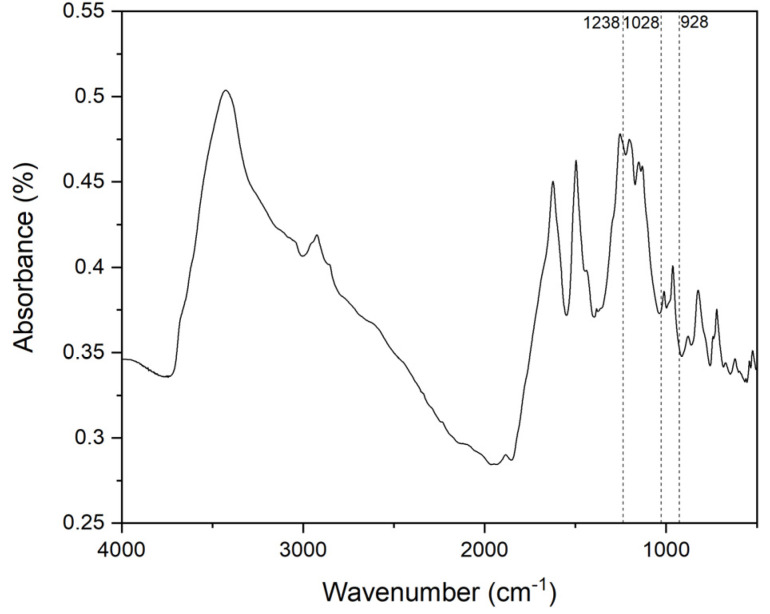
FT-IR spectrum of poly(BAF-oda-fu).

FT-IR was used again to characterize the polymerized sample, and to ensure the success of the polymerization method. The FT-IR spectrum of poly(BAF-oda-fu) is seen in Figure 6. As can be seen in the figure, the antisymmetric vibrations of C-O-C at 1238 cm^−1^ and the C-N band at 1028 cm^−1^ disappeared. Furthermore, the oxazine ring mode at 928 cm^−1^ disappeared as well, indicating the ring-opening reaction of the benzoxazine group [42]. A broad O-H stretching band appeared at 3000–3500 cm^−1^, which is expected for polybenzoxazines.

Out-gassing during resin polymerization was analyzed by TGA–FT-IR. The resins BAF-oda-fu and BAF-oda-fu-POSS were first dried under vacuum at room temperature for 48 h. Each resin was then heated in a TGA from room temperature to 200 °C over a time frame of approximately 60 min while the evolved gases were monitored by FT-IR. The BAF-oda-fu resin evolved chloroform and carbon dioxide. The BAF-oda-fu-POSS resin evolved carbon dioxide only. The release of chloroform is unsurprising because this was the solvent used in the synthesis of BAF-oda-fu. Further, the process for preparing the BAF-oda-fu-POSS also removed the chloroform. The source of the evolved carbon dioxide from both resins is believed to be from impurities such as reaction side-products.

### 3.3. Thermal Analysis 

**Figure 7:**
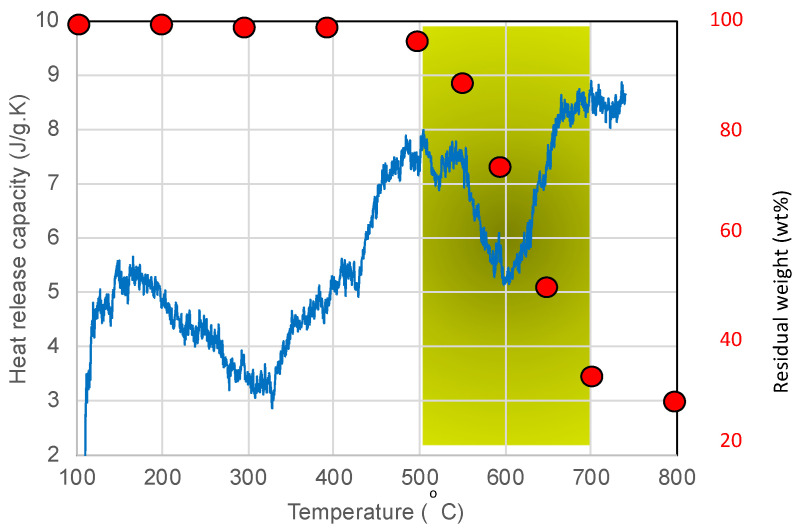
A microcombustion calorimetric (blue line) and TGA (red closed circles) thermograms of poly(BAF-oda-fu). The main thermal degradation temperature range is indicated by a green belt between 500 and 700 °C.

Thermal analysis of the polymer was performed using TGA. The TGA thermogram seen in Figure 7 shows a T_d5_ (temperature at 5% weight loss) and T_d10_ (temperature at 10% weight loss) of 418 °C and 465 °C, respectively. Both degradation temperatures are considered quite high. The char yield, on the other hand, is very low for benzoxazines, particularly for those containing a CF_3_ group, which is typically in the range of 36–68% [7,11,12,17,19,23,23,27,34], or a furan group, which is typically between 30–74% due to high crosslinking capabilities [41,43,44,45,46,47,48,49,50]. The char yield at 800 °C was approximately 24%, which is unexpectedly low for a benzoxazine such as BAF-oda-fu, which contains both above functionalities. The results indicate good thermal degradation resistance but poor char yield at very high temperatures. However, these temperatures (i.e., 800 °C) are not reached in low-Earth orbit, thus this is not a major concern for the desired application. For example, cyclic temperature variations for materials outside of the International Space Station generally range from −120 °C to +120 °C.

While a detailed analysis is needed to understand this unexpected result, it is hypothesized that the molecular mechanism of this behavior relates to the flame-retarding action of halogenated polymers. Weight reduction occurs upon the fragmentation of the polymer chain at a degradation temperature. Those molecular fragments with free radicals either undergo secondary reactions to stay as part of the crosslinked network or form the precursor for char. The rapid weight reduction of polytetrafluoroethylene also appears around 450 °C. This temperature coincides with the degradation temperature of the current fluorinated polybenzoxazine, which is in the range of 400–800 °C, but particular rapid degradation centers around 500–550 °C. Thus, it is expected that the fluorine free radicals are readily available to terminate the free radicals of the degraded molecular fragments of the benzoxazine networks during the degradation process of poly(BAF-oda-fu). This secondary reaction discourages the formation of char and maintains the volatility of the molecular fragments. This hypothesis is consistent with two observations, namely the non-combustible nature and low char yield. The van Krevelen equation shown below is widely used to predict the limiting oxygen index (LOI) value [51].

(2) LOI(%)=0.4CR+17.5

where CR is the char residue (char yield in %) after heating to 850 °C under nitrogen.

Although there are a few different values proposed, materials with an LOI value above 28 can be considered self-extinguishing [52]. Certainly, the current polymer would have one of the highest LOI values reported to date judging from the heat release capacity value being one of the smallest reported, as shown in Figure 7. A heat release capacity of less than 10 represents one of the lowest values of all polymers ever reported. Although further detailed study is needed to understand the complex flaming behavior observed, the polymer is clearly a non-combustible polymer since a heat release capacity less than 100 is considered to be non-combustible [53].

However, the LOI value calculated from the van Krevelen equation is 25.5 using the extrapolated char yield value of 20% at 850 °C. Thus, from the calculation, the polymer would not be considered as self-extinguishing, not to mention non-combustible. Accordingly, the van Krevelen equation may not be applicable for those polymers with multiple degradation mechanisms such as the one observed with the current fluorinated polybenzoxazine where molecular fragments are thought to be terminated by fluorine free radicals, leading to volatile secondary molecules, rather than leading to non-volatile char formation via secondary reactions. Brominated molecules are commonly used as halogenated flame retardants. It is important to realize that this is not because bromine is an unusually effective flame retardant among other halogen atoms, but because the C-Br bond-cleavage temperature roughly corresponds with the degradation temperature of many polymers that are desired to be flame-retarded, such as epoxy resins used in the printed circuit board industry. However, if the polymer to be flame-retarded degrades at a much lower temperature than the epoxy resin, then iodine may be more effective. On the contrary, if the polymer degrades at a much higher temperature, then fluorine may be more effective, as in the example in this paper. Thus, it is the similarity of the C-X (X being a halogen atom) cleavage temperature and polymer degradation temperature that is critical for the choice of the most effective halogenated flame retardant.

### 3.4. Dynamic Mechanical Analysis of Composite Samples

**Figure 8:**
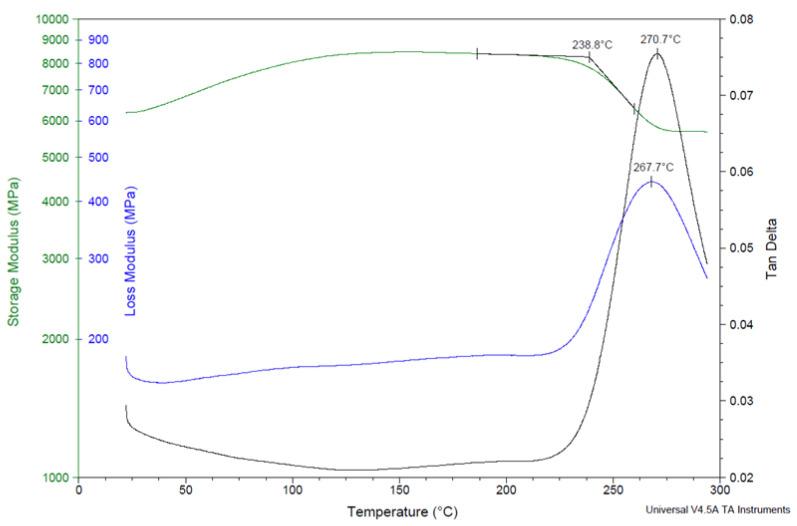
DMA thermogram for Nextel 312/poly(BAF-oda-fu) composite with no post-cure heat treatment.

Dynamic mechanical analysis was performed on composite samples to evaluate the glass transition temperature (Tg) and maximum storage modulus (E’_max_). The results are reported in Table 1 for three different composite samples. The heat treatment of the composite and the addition of POSS to the matrix resin were both shown to increase the Tg. The DMA Tg of the Nextel 312/poly(BAF-oda-fu) sample with no heat treatment was determined to be 239 °C. The DMA thermogram for this sample is provided in Figure 8 below. The heat-treated Nextel 312/poly(BAF-oda-fu) sample exhibited a DMA Tg of 273 °C, which is a 34 °C increase over the sample with no heat treatment. The DMA Tg for the heat-treated sample of Nextel 312/poly(BAF-oda-fu-POSS) was found to provide the highest DMA Tg of the three samples at 308 °C. This sample did not exhibit a tan δ peak before the test was stopped at 450 °C.

E’_max_ was investigated to evaluate composite stiffness. All of the samples experienced their maximum E’ values at elevated temperatures, potentially indicating that additional crosslinking was occurring as the samples were heated during the DMA tests. Heat treatment and the addition of POSS to the matrix resin were shown to increase E’_max_. The Nextel 312/poly(BAF-oda-fu) sample with no heat treatment exhibited an E’_max_ value of 8470 MPa at 153 °C. An increase of 48% in E’_max_, to 12,550 MPa, was achieved for the heat-treated Nextel 312/poly(BAF-oda-fu-POSS) sample.

### 3.5. Density Measurements and Composite Void Analysis

The density of poly(BAF-oda-fu) polybenzoxazine was found to be 1.38 g/cm^3^, which is significantly higher than typical polybenzoxazines. The higher density is believed to be due to the fluorine content in the polymer.

The composite specimens were found to have densities ranging from 1.61 to 1.77 g/cm^3^. This range is below the theoretical density of a void-free composite comprised of 30 wt% poly(BAF-oda-fu) and 70 wt% Nextel 312 reinforcement. An analysis was performed to estimate the void content in the composite specimens following the guidance of ASTM D2734 (Standard Test Methods for Void Content of Reinforced Plastics). The composites were found to have a void content of greater than 15 vol%. There are several potential contributing factors to the high void content, including out-gassing of the resin during curing, the use of thick fabric, and the relatively low pressure of the vacuum bagging process.

### 3.6. Ground-Based Atomic-Oxygen Erosion Testing

Ground-based atomic-oxygen erosion testing was performed on the composite samples to evaluate their erosion resistance when exposed to an atomic-oxygen-rich environment. Testing was conducted in accordance with ASTM E2089 (Standard Practices for Ground Laboratory Atomic-Oxygen Interaction Evaluation of Materials for Space Applications). Two samples of Nextel 312/poly(BAF-oda-fu) composite and two samples of Nextel 312/poly(BAF-oda-fu-POSS) composite were tested. Testing was conducted in a 60-Watt RF plasma etcher/asher (Model PE-250) under a vacuum of 100 Torr for a total of 37 h. Kapton HN film with a thickness of 127 μm (5 mil) was used as the witness material to determine the effective atomic-oxygen fluence during the test.

The atomic-oxygen flux within a plasma asher varies based on the sample position. Therefore, a separate calibration test was conducted using Kapton HN film in all sample holder positions to determine the flux distribution within the plasma asher. Using the calibration values, the effective fluence for each sample was adjusted based on its position in the sample holder.

**Figure 9:**
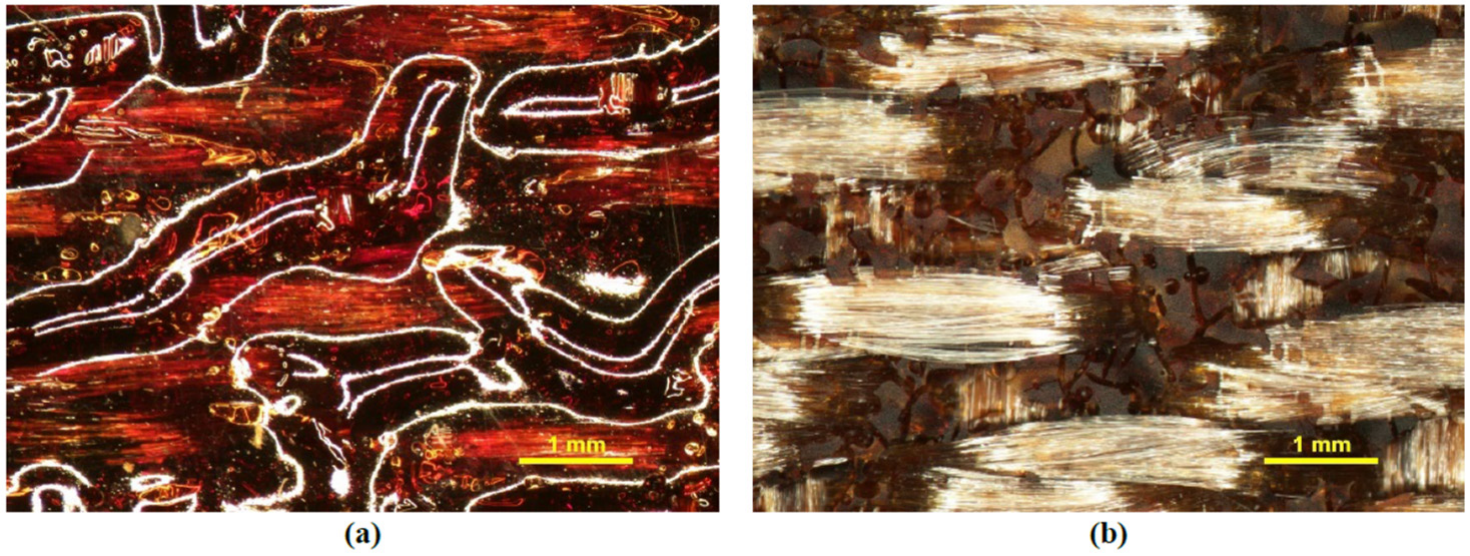
Optical microscopy images comparing Nextel 312/poly(BAF-oda-fu) composite surface features (**a**) before and (**b**) after ground-based atomic-oxygen erosion testing.

**Figure 10:**
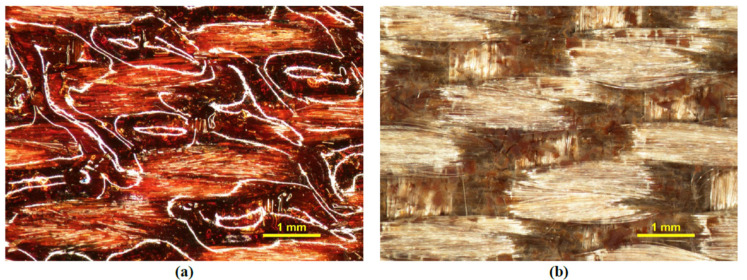
Optical microscopy images comparing Nextel 312/poly(BAF-oda-fu-POSS) composite surface features (**a**) before and (**b**) after ground-based atomic-oxygen erosion testing.

The ground-based atomic-oxygen erosion testing results are provided in Table 2 below. The average atomic-oxygen erosion yields for Nextel 312/poly(BAF-oda-fu) and Nextel 312/poly(BAF-oda-fu-POSS) were 4.32 and 4.00 cm^3^/atom × 10^−24^, respectively. The addition of the POSS additive improved the atomic-oxygen resistance (reduced atomic-oxygen erosion yield). Optical microscopy of the composite surface after atomic-oxygen exposure was performed using a Nikon SMZ1000 stereomicroscope. Representative before and after images for the two types of composites are provided below in Figure 9 and Figure 10. (The “before” micrographs exhibit numerous white curved features due to light reflections off the very glossy surface of the resin.) The microscopy revealed that portions of the polymer matrix were removed by erosion. The alumina fabric, however, did not show obvious signs of erosion. This is not surprising given the fact that alumina fibers have a very high resistance to atomic oxygen. Erosion yield values less than 0.025 × 10^−24^ cm^3^/atom have been reported in the literature for alumina [54]. Thus, it is estimated that the erosion yield values reported for the composite samples are primarily due to contributions from the polymer matrix and not the reinforcing fibers. The alumina fibers did not form a solid barrier that protected the exposed surface of the composite. Rather, the fibers functioned primarily as mechanical reinforcement while providing some localized protection for the polymer matrix.

The ground-based testing results for poly(BAF-oda-fu) were compared to testing results for nine fluorinated polymers reported in the literature by NASA to provide perspective in terms of erosion resistance performance. The nine fluorinated polymers were previously tested by NASA using a ground-based RF plasma asher and in space during Materials International Space Station Experiment (MISSE) 2. Atomic-oxygen erosion yield and estimated fluorine concentration were used as performance metrics, provided in Table 3. The erosion yield value listed for poly(BAF-oda-fu) is estimated to be the same as that of the Nextel 312/poly(BAF-oda-fu) composite since the erosion is dominated by the polymer matrix and not the alumina fiber reinforcement.

**Figure 11:**
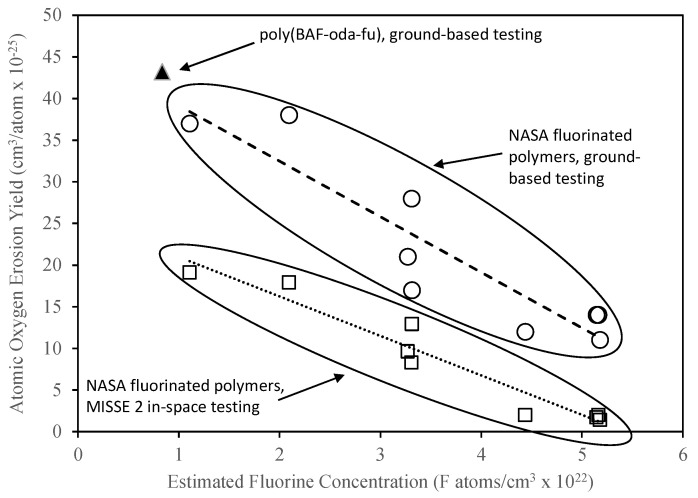
Plot of atomic-oxygen erosion yield vs. estimated fluorine concentration for poly(BAF-oda-fu) and selected fluorinated polymers.

Figure 11 provides a plot of atomic-oxygen erosion yield versus fluorine concentration for all of the polymers listed in Table 3. The ground-based testing and in-space testing results are grouped separately because the ground-based results differ consistently from the in-space results. As seen from the plot, a reasonably linear relationship exists between atomic-oxygen erosion yield and fluorine concentration for both groups of test results. The in-space testing produced lower atomic-oxygen erosion yields due to the many differences between the ground-based testing environment in the RF plasma asher and the actual space environment in low-Earth orbit. Poly(BAF-oda-fu) is shown to align well with the linear trend line of the NASA ground-based testing group. Poly(BAF-oda-fu) would be expected to have a significantly lower atomic-oxygen erosion yield in low-Earth orbit, following the trend of the other materials. It is interesting to note that the contribution of the polymer structure had only a minor contribution to the atomic-oxygen erosion resistance, and the main factor was the polymer’s fluorine content per unit volume. Polybenzoxazine has considerably different structures and properties than those polymers evaluated by NASA, yet it fell in a general linearity trend. This suggests that a polymer having good molecular design flexibility to incorporate more fluorine atoms in its structure will show higher atomic-oxygen erosion resistance.

## 4. Conclusions

A benzoxazine monomer was synthesized from bisphenol AF, 4,4′-oxydianiline, furfurylamine, and paraformaldehyde, to be used in low-Earth orbit as an atomic-oxygen-resistant material. Successful preparation was achieved via a 12 h synthesis in chloroform. A combination of 1N aq. NaOH and water washes followed by purification via column chromatography was successful in eliminating impurities from the product, resulting in a technical grade (95% pure) resin. The monomer was characterized via ^1^H NMR, FT-IR, and DSC analyses. The polymerization temperature was found to be approximately 238 °C, a typical value for polybenzoxazines. The polymerized samples were further analyzed with TGA, which demonstrated the high T_d5_ and T_d10_ of poly(BAF-oda-fu). However, the TGA also showed that the char yield at 800 °C was low, though a working hypothesis suggests this is due to the flame-retardant capabilities of the fluorinated polymer. Composites with alumina fiber reinforcement were prepared and characterized. The composites exhibited glass transition temperatures as high as 308 °C, depending on the utilization of the POSS additive and heat treatment. The atomic-oxygen resistance was consistent with expectations based on the fluorine content of the polymer.

## Figures and Tables

**Table 1:** Summary of Dynamic Mechanical Analysis Results.

Sample	Cure Schedule	Post-Cure Heat Treatment	E’_max_	Glass Transition Temperature		
				DMA T_g_	E” Peak	Tan δ Peak
Nextel 312/ poly(BAF-oda-fu) composite, no heat treatment	2 h at 200 °C	None	8470 MPa (at 153 °C)	239 °C	268 °C	271 °C
Nextel 312/ poly(BAF-oda-fu) composite, heat treated	2 h at 200 °C	30 min at 240 °C	10670 MPa (at 261 °C)	273 °C	299 °C	310 °C
Nextel 312/ poly(BAF-oda-fu-POSS) composite, heat treated	2 h at 200 °C	30 min at 240 °C	12550 MPa (at 254 °C)	308 °C	336 °C	Peak not observed

**Table 2:** Summary of Ground-Based Atomic-Oxygen Erosion Testing.

Material	Sample ID	Density (g/cm^3^)	Surface Area (cm^2^)	Mass Loss (g)	Effective Fluence (atoms/cm^2^ × 10^21^)	Ground-Based Atomic Oxygen Erosion Yield (cm^3^/atom × 10^−24^)
Nextel 312/poly(BAF-oda-fu) composite, heat treated	A1	1.61	8.64	0.1353	2.20	4.43
	A2	1.68	8.85	0.1440	2.30	4.20
Nextel 312/poly(BAF-oda-fu-POSS) composite, heat treated	B1	1.64	8.73	0.1120	1.99	3.93
	B2	1.65	8.79	0.0923	1.57	4.06

**Table 3:** Physical Properties, Atomic-Oxygen Erosion Yields, and Estimated Fluorine Concentration for poly(BAF-oda-fu) and Select Fluorinated Polymers Previously Tested by NASA.

Polymer Type	Repeat Unit	Molar Mass (g/mol)	Density (g/cm^3^)	Atomic Oxygen Erosion Yield (cm^3^/atom × 10^−25^)		Estimated Fluorine Concentration (F atoms/cm^3^ × 10^22^)
				Ground-Based RF Plasma Asher	In-Space MISSE 2	
Poly(BAF-oda-fu)	C_62_H_54_F_12_N_4_O_7_	1195.09	1.38	43.2*	Not Tested	0.83
Polyimide (fluorinated)	C_46_H_22_O_6_N_2_F_12_	926.66	1.42	37.0	19.1	1.11
Ethylene-chlorotrifluoroethylene	C_4_H_4_ClF_3_	144.52	1.68	38.0	17.9	2.10
Ethylene-tetrafluoroethylene	C_4_H_4_F_4_	128.07	1.74	21.0	9.6	3.27
Chlorotrifluoroethylene	C_2_ClF_3_	116.47	2.13	28.0	8.3	3.31
Polyvinylidene fluoride	C_2_H_2_F_2_	64.03	1.76	17.0	12.9	3.31
Amorphous fluoropolymer	C_19_O_6_F_32_	932.15	2.15	12.0	2.0	4.44
Perfluoroalkoxy copolymer resin	C_203_OF_406_	10167.52	2.14	14.0	1.7	5.14
Fluorinated ethylene propylene	C_11_F_22_	550.08	2.14	14.0	2.0	5.16
Polytetrafluoroethylene	C_2_F_4_	100.02	2.15	11.0	1.4	5.18

## Data Availability

Data presented in this study are available on request from the corresponding author.
